# A retrospective clinical analysis of 11 cases of PEComa from different sites

**DOI:** 10.1186/s12957-024-03349-x

**Published:** 2024-04-30

**Authors:** Jinbowen Yan, Dan Zhou, Yifei Wang, Bowen Yang, Yuefeng Wang, Kaili Zhang, Shuo Zhang, Bo Zhang, Qingwei Meng, Qiubo Lv

**Affiliations:** 1 Department of Obstetrics and Gynecology, Beijing Hospital, National Center of Gerontology, Institute of Geriatric Medicine, Chinese Academy of Medical Sciences, Beijing, 100730 P. R. China; 2 Department of Pathology, Beijing Hospital, National Center of Gerontology, Institute of Geriatric Medicine, Chinese Academy of Medical Science, Beijing, 100730 P. R. China; 3 Department of Radiology, Beijing Hospital, National Center of Gerontology, Institute of Geriatric Medicine, Chinese Academy of Medical Sciences, Beijing, 100730 P. R. China; 4 The Key Laboratory of Geriatrics, Beijing Institute of Geriatrics, Institute of Geriatric Medicine, Beijing Hospital, Chinese Academy of Medical Sciences, National Center of Gerontology of National Health Commission, Beijing, P. R. China

**Keywords:** Perivascular epithelioid cell tumors, PEComa, Clinical features, Pathogenesis, Treatment strategies

## Abstract

**Purpose:**

The objective of this paper is to offer a thorough examination of the clinical presentations, etiology, and treatment strategies associated with perivascular epithelioid cell tumors (PEComas).

**Methods:**

This retrospective study examined the comprehensive archival data of PEComa cases diagnosed at Beijing Hospital from 2015 to 2023. The pathology slides of all patients were thoroughly reassessed by two experienced pathologists. A thorough retrospective analysis was undertaken, incorporating clinicopathological data including gender, age at diagnosis, initial clinical manifestations, signs, disease onset site, tumor markers, imaging findings, therapeutic modalities, pathological features, immunohistochemical profiles, treatment responses, and prognostic indicators. Patients were evaluated for disease severity according to established pathological classification criteria and were followed up until the designated analysis cut-off date. In instances where patients were unable to be monitored on-site, they were contacted via telephone for postoperative follow-up inquiries.

**Results:**

This study included 11 patients with ages ranging from 17 to 66 years old, presenting with the disease in multiple anatomical sites, including the retroperitoneum (2/11), liver (4/11), kidney (4/11), lung (1/11), and broad ligament of the uterus (1/11). Most patients presented with non-specific clinical symptoms and were subsequently diagnosed with space-occupying lesions upon physical examination. The tumor demonstrated progressive growth and enlargement, which could result in compression of neighboring organs. Preoperative imaging alone is insufficient for a definitive diagnosis of PEComa, but MRI can provide an initial evaluation of the tumor’s potential malignancy. Molecular marker testing specific to PEComa, such as HMB-45 (90.0%), SMA (81.8%), Melan-A (90.9%), vimentin (90.9%), and Desmin (36.3%), was conducted on all patients. No adjuvant therapies were administered postoperatively. Upon analysis, no instances of relapse at the primary site or the development of new tumors at other sites were observed. Regular imaging reviews of three patients with malignant PEComa post-surgery showed no evidence of recurrence.

**Conclusions:**

The clinical presentation, tumor biomarkers, and imaging characteristics of PEComa lack specificity, necessitating dependence on pathology and immunohistochemistry for precise diagnosis. The mainstay of treatment consists of surgical resection, with patients typically experiencing a favorable prognosis.

## Introduction

Perivascular epithelioid cell tumors (PEComas) are a group of mesenchymal tumors identified by the World Health Organization (WHO) as having distinct perivascular cells [1]. This category predominantly includes angiomyolipoma (AML), pulmonary clear cell “sugar” tumor, and lymphangioleiomyomatosis (LAM) [2]. Although the uterus is a frequent site of PEComa development, these tumors can also arise in diverse locations such as the kidney, pancreas, urinary bladder, uterus, and liver [3, 4]. The majority of cases of PEComa present as benign lesions, with no universally accepted protocol for the diagnosis and treatment of treatment-naïve, recurrent, and malignant PEComa globally. Surgical resection continues to be the primary clinical treatment approach [5]. This study involved a retrospective analysis of 11 patients with pathologically confirmed PEComa at different sites of origin. The objective of this study was to synthesize and analyze the clinical features, pathogenesis, and treatment approaches of PEComas in order to enhance the existing knowledge and therapeutic practices.

## Patient and methods

### Patients

We employed the search terms ‘Perivascular epithelioid cell tumors’ and ‘PEComas’ to retrieve the pathological diagnoses of patients at Beijing Hospital from 2015 to 2023 within the pathology system. Two experienced pathologists conducted a comprehensive reassessment of all pathology sections obtained from the patients. Subsequently, we compiled and retrospectively reviewed the complete case files within the case system. The study was approved by the ethics committee of Beijing Hospital (no.2019BJYYEC-250-02).

### Clinical data

A thorough examination was carried out, incorporating clinicopathological data including sex, age at diagnosis, initial clinical symptoms, signs, location of disease onset, tumor markers, imaging results, treatment approaches, pathological characteristics, immunohistochemistry findings, treatment outcomes, and prognosis. Disease severity was evaluated according to established pathological classification criteria, and patients were followed up until the specified analysis cut-off date. In cases where patients were unable to maintain ongoing follow-up at our facility, attempts were made to collect postoperative follow-up data through telephone correspondence. Telephone follow-up was conducted with the patient’s explicit consent.

## Result

This study involved a cohort of 11 patients aged between 17 and 66 years. Of these patients, seven were female and four were male. The disease was observed in multiple anatomical sites, including the retroperitoneum (2/11), liver (4/11), kidney (4/11), lung (1/11), and broad ligament of the uterus (1/11) as shown in Table 1.

**Table 1:** Clinical Pathology Information

Number	Sexual	Age	Site	Subtype	Maximum cross-section(cm)	Neoplastic nature	Follow up (months)	Prognosis
1	F	43	The right broad ligament	AML	18.9	Low malignant	38.1	Nonrelapse
2	F	66	The upper portion of the left kidney	AML	3.1	Benign	91.8	Nonrelapse
3	F	41	The lower pole of the left kidney	AML	1	Benign	32.3	Nonrelapse
4	F	44	The middle of the right kidney	AML	2.8	Benign	31.0	Nonrelapse
5	M	65	Hepatic segments V, VI and VIII	AML	6.7	Benign	3.1	Nonrelapse
6	F	36	Hepatic segments VI	AML	3.3	Benign	41.6	Nonrelapse
7	F	36	Hepatic segments VI, VII and VIII	AML	4.8	Benign	8.4	Nonrelapse
8	F	34	Left lateral liver	AML	3.1	Benign	4.9	Nonrelapse
9	M	62	Lower lobe of the right lung	pulmonary clear cell “sugar” tumor	2.5	Benign	3.2	Nonrelapse
10	M	30	Retroperitoneal (RP)	AML	16	Malignant	69.8	Nonrelapse
11	M	17	RP	AML	19.5	Moderate malignant	4.6	Nonrelapse

F: female M: male

### Clinical manifestations

The majority of patients presented with asymptomatic space-occupying lesions upon physical examination, indicative of gradual tumor growth and potential compression of adjacent organs. Within the study population, 27.2% of patients reported experiencing pain localized to the lower back or abdomen. Despite the tumor location, liver and kidney function remained within normal limits. In a single patient presenting with retroperitoneal involvement and partial kidney disease, the CA125 level was found to be slightly elevated at 37.8 U/mol. A separate patient with liver disease displayed a CA199 level of 93.9 U/mol, whereas individuals with extensive uterine ligament involvement exhibited a CA125 level of 44.4 U/mol.

### Radiographic examination

**Fig. 1:**
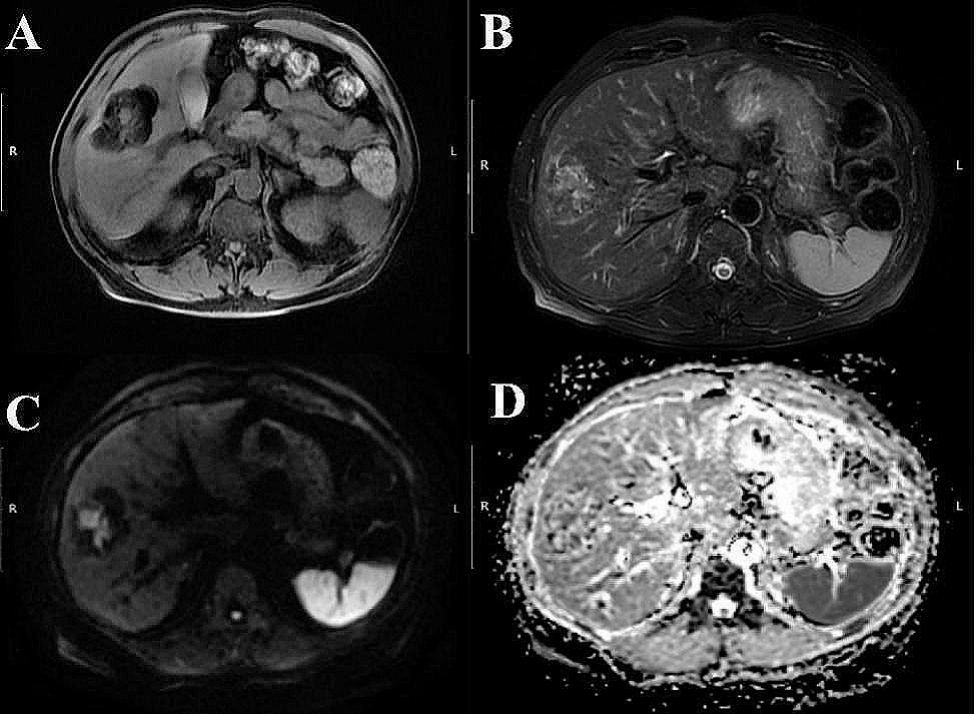
The MRI image depicts a benign hepatic PEComa, characterized by a round abnormal signal shadow measuring approximately 5.2 cm×6.7 cm×4.7 cm in the right lobe of the liver. This shadow appears lobulated and well defined, exhibiting predominantly mature fatty signal with interspersed masses of soft tissue signal. The image panels **A**, **B**, **C**, and **D** represent T1WI, T2WI, DWI, and ADC images, respectively

PEComa poses a challenge in terms of detection on CT and MRI scans, leading to a restricted diagnostic utility of imaging modalities. CT scans commonly depict a distinct, round or quasicircular soft tissue mass with uniform density, although variations in internal density may occur due to factors such as fat, necrotic cystic changes, hemorrhage, and occasional calcifications. In contrast, MRI findings are often complex and varied due to the presence of unique internal components within the tumor. Lesions can demonstrate diverse features, including mature fat deposition (fatty degeneration), necrotic cystic alterations, and hemorrhage. Moreover, larger lesions may manifest short pseudocapsules along their periphery. The tumor commonly exhibits characteristics such as decreased signal intensity on T1-weighted imaging (T1WI), variable signal intensity on T2-weighted imaging (T2WI), increased signal intensity on diffusion-weighted imaging (DWI), decreased signal intensity on apparent diffusion coefficient maps (ADC), and moderate to marked enhancement during the arterial phase post-contrast administration. Additionally, there is a possibility of a minor reduction in contrast enhancement during the venous and delayed phases, as well as the potential presence of dotted or linear vascular shadows and delayed enhancement pseudocapsules in specific lesions. (Fig. 1).

**Fig. 2:**
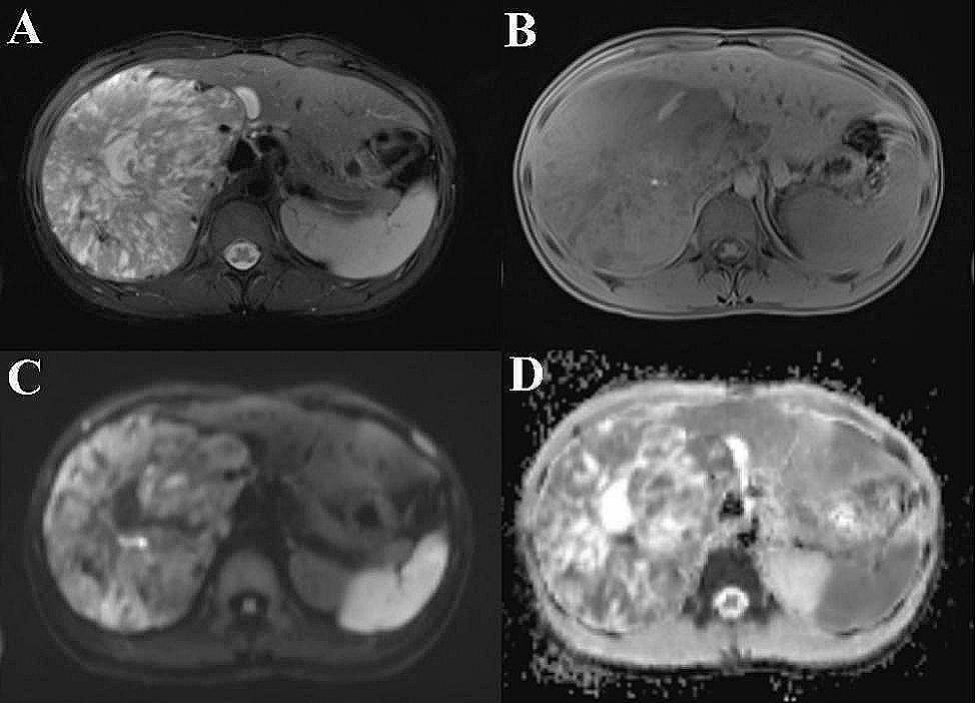
The provided figures depict an MRI scan showcasing a moderately malignant PEComa located in the right retroperitoneum. The image panels **A**, **B**, **C**, and **D** represent T1WI, T2WI, DWI, and ADC images, respectively

Preoperative imaging techniques do not provide a definitive diagnosis of PEComa; however, magnetic resonance imaging (MRI) can provide an initial assessment of both benign and malignant tumors. The MRI findings indicated the presence of a substantial retroperitoneal mass on the right side, characterized by well-defined margins and dimensions measuring approximately 19.5 × 12.4 × 13.5 cm. Signal intensities within the mass were varied, with a predominance of slightly low signal intensity on T1-weighted imaging. Significantly, an irregular necrotic region with a striped high signal shadow was identified at the central aspect of the mass. T2-weighted imaging demonstrates a confluence of high signal intensity and the existence of numerous vascular flow voids. Furthermore, an elevation in signal intensity within the parenchyma of the mass is noted on diffusion-weighted imaging. The right kidney exhibits medial and inferior displacement, with an indistinct demarcation between its upper pole and the mass. Moreover, the parenchyma of the right kidney displays a claw-like appearance at the border with the mass, the right adrenal gland shows indistinct boundaries, and the right renal sinus demonstrates enlargement and thickening. The identified mass is considered malignant and is likely of renal origin. (Fig. 2).

### Pathological diagnosis

**Fig. 3:**
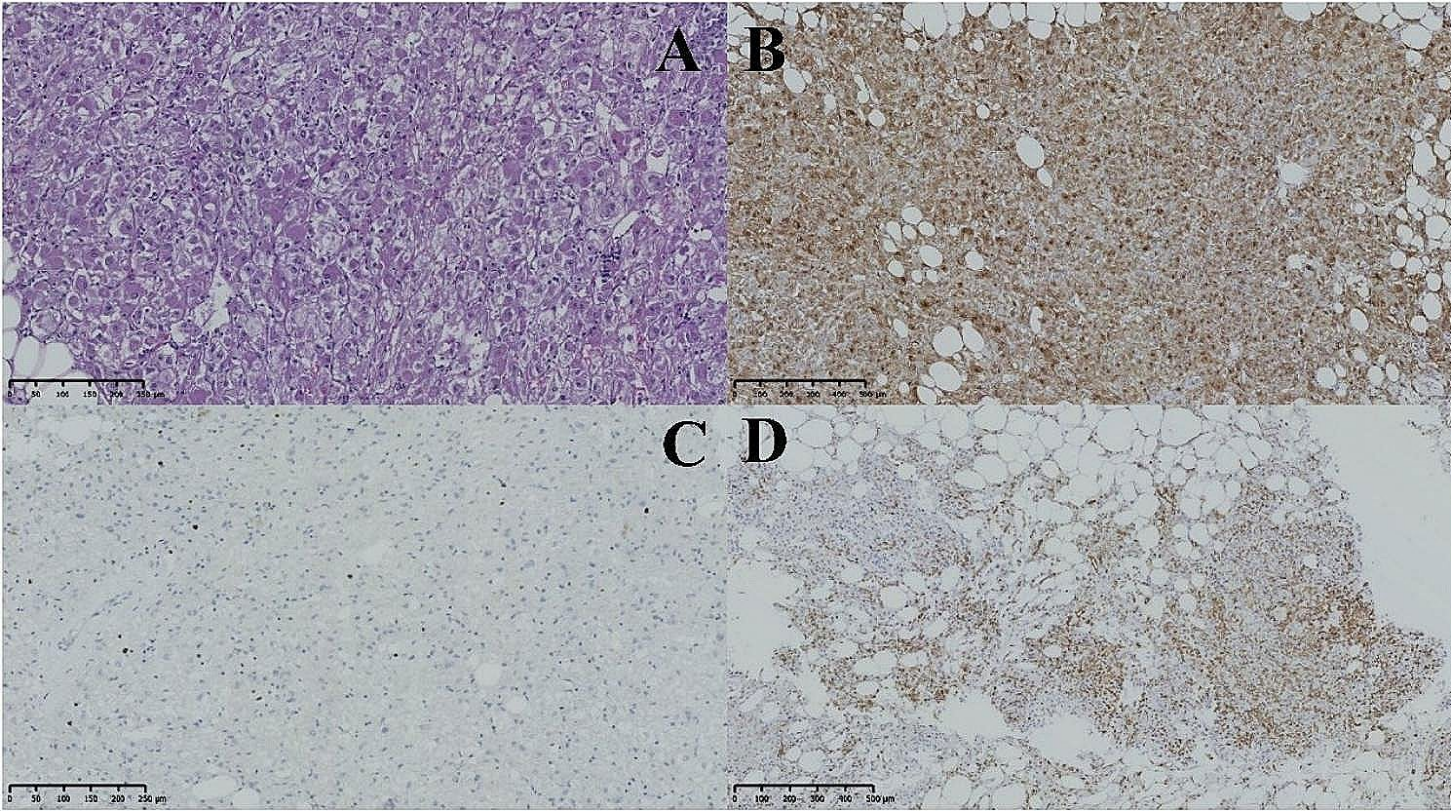
The provided figures depict pathological images of a benign hepatic PEComa. Figure A illustrates tumor cells exhibiting mild atypia at a magnification of ×200. Figure B demonstrates HMB45 positivity in tumor cells at a magnification of ×100. Figure C displays 5% positivity of Ki-67 in tumor cells at a magnification of ×200. Lastly, Figure D exhibits vimentin flecked positivity in tumor cells at a magnification of ×100

**Fig. 4:**
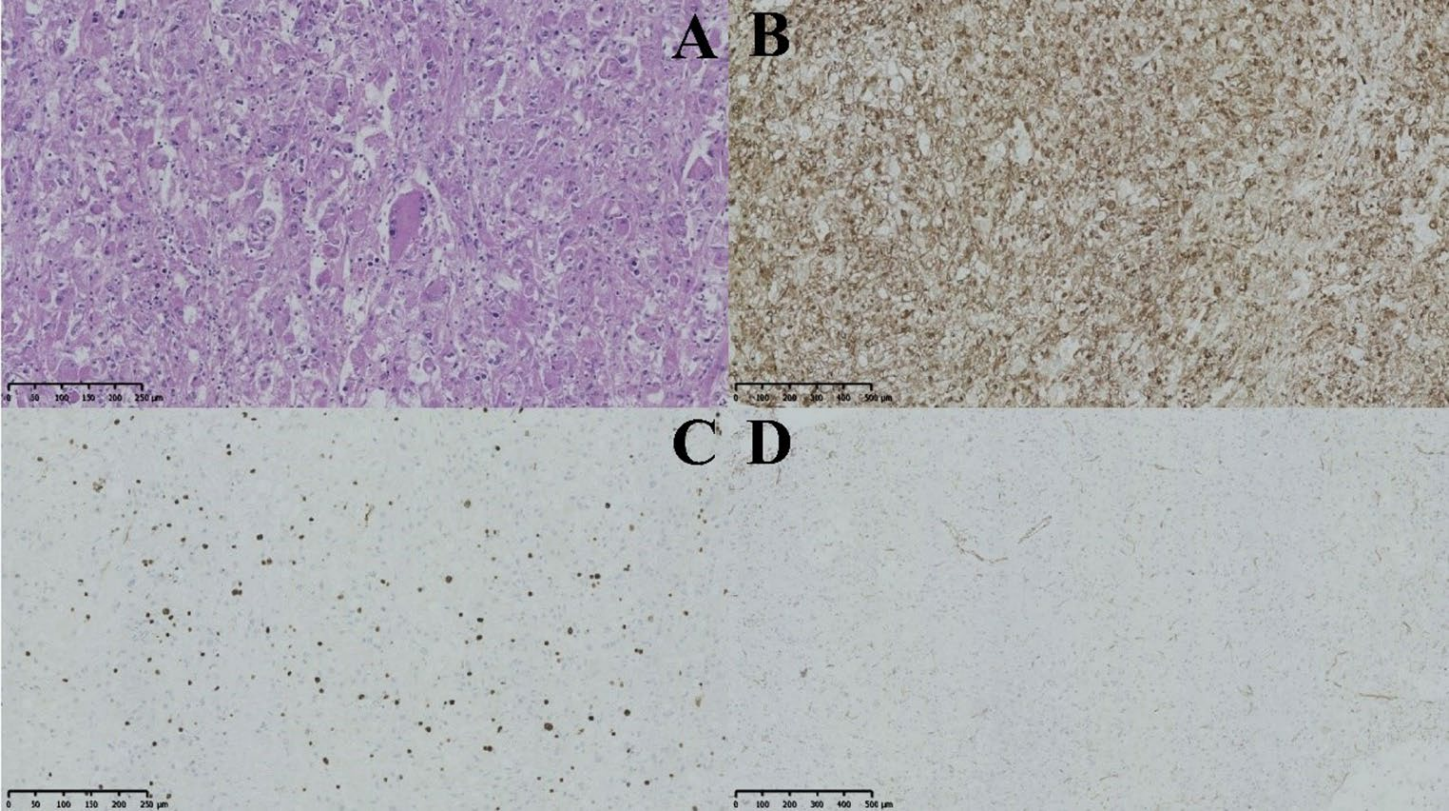
The provided images depict a retroperitoneal PEComa with moderate malignancy. Image A reveals tumor cells with moderate atypia and multinucleated tumor giant cells at a magnification of 200x. Image B demonstrates HMB45 positivity in tumor cells at a magnification of 100x. Image C displays 20% positivity of Ki-67 in tumor cells at a magnification of 200x. Lastly, image D exhibits the absence of Vimentin in tumor cells at a magnification of 100x.

The eight benign lesions displayed a consistent histological pattern characterized by the arrangement of tumor cells in nests or sheets, with radiolucent or clear cytoplasm surrounding vessels and round or ovoid nuclei with nucleoli (see Fig. 3). Among the cases analyzed, three were determined to be malignant, including a retroperitoneal malignant PEComa with a tumor size of approximately 16 × 11 × 11 cm. In this particular case, the tumor cells exhibited basophilic cytoplasm, abundant cytoplasmic content, nuclear heterogeneity, a higher incidence of local nuclear divisions (4 out of 10 high-power fields), visible focal necrosis, and a local Ki-67 index of 5%. Additionally, another instance of moderately malignant retroperitoneal PEComa was identified, distinguished by the presence of 7–8 nuclear schizograms per high-power field and observable multinucleated tumor giant cells displaying hemorrhage and necrosis. The tumor invaded the renal parenchyma, achieving a maximum diameter of 17.5 cm, with no evidence of tumor involvement at the renal margin. The FNCLCC score was assessed as 4. In instances of low-grade malignant PEComa originating from the uterine ligament, some tumors exhibited individual sizes exceeding 10 cm in diameter. The cells predominantly displayed epithelioid characteristics, showing heterogeneity and 1–3 nuclear divisions per high-power field, with no apparent necrosis. (Fig. 4).

All participants in the study were subjected to testing for particular molecular markers linked with PEComa [6], such as HMB-45 (90.0%), SMA (81.8%), Melan-A (90.9%), vimentin (90.9%), and Desmin (36.3%). The immunohistochemical markers CD117, S100, and inhibin were found to be negative in the study. Among the patient cohort, three individuals tested positive for TFE3, with one case of benign liver PEComa, one case of benign right pulmonary PEComa, and one case of malignant retroperitoneal PEComa. Furthermore, all 11 patients demonstrated positive Ki-67 staining. The results of the immunohistochemical analysis for the 11 cases are summarized in Table 2.

**Table 2:** Immunohistochemistry

Immunohistochemical Marker	Positive Rate(%)
HMB45	90.9
SMA	81.8
Melan-A	90.9
Vimentin	90.9
Desmin	36.3
TFE3	27.3

#### Treatment and prognosis

All patients received surgical intervention without subsequent complementary therapies. Upon analysis, no instances of relapse at the primary site or the development of new tumors at secondary locations were observed. Three patients diagnosed with malignant PEComa underwent regular post-operative evaluations, with imaging examinations showing no signs of recurrence.

## Discussion

PEComa is a rare medical condition characterized by the presence of melanin and myogenic markers. It has been observed to occur in various anatomical locations, with a predilection for the kidneys [7]. Additionally, cases of PEComa have been reported in the liver, pancreas, rectum, abdomen, gynecological urethra, and other sites [8–11]. This study involved the analysis of clinicopathological data from 11 patients diagnosed with PEComa, including those with involvement in the aforementioned common sites of disease. Notably, there is a significant female predominance in the incidence of PEComas, with a female-to-male ratio of 6:1. These tumors have been observed to occur across all age groups, with a higher prevalence among young to middle-aged adults [12]. The study included a total of eleven participants, consisting of seven females and four males, demonstrating a varied distribution in age. It is worth mentioning that the subgroup of patients diagnosed with malignancy belonged to a relatively younger age group.

PEComa lacks a specific clinical presentation, even in instances of malignant pathological diagnosis. Ultrasound imaging is effective in detecting tumors in various anatomical locations such as the liver, kidney, retroperitoneum, and uterus. As the tumor grows in size, it may compress adjacent organs, resulting in the development of symptoms. Patients may experience varying degrees of pain, with the intensity potentially linked to the size and location of the tumor. Notably, tumors larger than 3 cm may cause persistent and vague discomfort, albeit manageable. Tumors situated in the kidney may present with symptoms of fatigue and back pain, along with manifestations such as waist pain, fatigue, and discomfort. Conversely, lesions in the pelvis have the potential to interfere with the regular menstrual cycle, particularly in instances of larger tumors that can elicit pain. Individuals suffering from liver disease may encounter symptoms such as abdominal pain, nausea, vomiting, and a range of other manifestations. However, a solitary patient with an extensive uterine ligament demonstrated a prolonged medical history marked by a gradual increase in menstrual volume. The pelvic mass achieved a maximum diameter of 16 cm, resulting in symptoms suggestive of bladder compression. Simultaneously, the patient’s liver function, kidney function, and blood count may remain within typical parameters. While tumor markers such as CA125 and CA199 may show a slight increase, their diagnostic accuracy is constrained. This investigation revealed that PEComa does not possess distinctive tumor markers. The study found that benign cases had a maximum CA199 level of 93.9 U/ml, while malignant cases had a maximum CA125 level of 44.4 U/ml. As a result, the use of tumor markers for assessing patients’ conditions during postoperative follow-up is considered to be unreliable.

The accurate preoperative imaging assessments present difficulties in diagnosing PEComa, highlighting the need to rely on pathological findings for confirmation. Preoperative imaging is unable to definitively diagnose PEComa; however, it can preliminarily determine the tumor’s benign or malignant characteristics, assist in localizing its location, and ascertain its size. This information is essential for surgical planning and determining the extent of the procedure. In this specific instance, preoperative MRI correctly identified all three cases of malignant PEComa.

The diagnosis of PEComa relies primarily on pathological evaluation, with lesions classified as benign, of uncertain malignant potential, or malignant. In this study, three cases were determined to be malignant. To differentiate between benign and malignant cases, Schoolmeester JK et al. [13] proposed an improved classification method inspired by the approach originally proposed by Folpe et al. [14]. The revised classification method for gynecological PEComa tumors incorporates five statistically significant characteristics: size (> 5 cm), high atypicality, > 1/50HPFs mitosis, necrosis, and lymphovascular invasion. The immunoreactivity of HMB45, SMA, Melan-A, Vimentin, and Desmin in PEComas has been extensively discussed, along with the progression of TFE3 staining positivity in PEComas. TFE3 is a member of the MiTF family of transcription factors, which includes MiTF, TFEB, TFEC, and TFE3 [15]. Approximately 20% of patients with PEComa demonstrate positive staining for TFE3, with a notable subset exhibiting TFE3 gene rearrangements [16]. Our investigation identified a prevalence of 27.2% of subjects displaying positive TFE3 staining. Previous studies suggest that PEComa cases associated with TFE3 may be indicative of a more aggressive disease trajectory or unfavorable prognosis [17]. Mammalian target of rapamycin (mTOR) inhibitors have shown limited therapeutic effectiveness in treating malignant PEComa with TFE3 rearrangement, whereas inhibitors targeting the vascular endothelial growth factor (VEGF)/VEGFR pathway offer a promising alternative therapeutic approach [18].

PEComas should be distinguished from mesenchymal neoplasms that display melanocyte expression, such as smooth muscle or melanocyte-differentiated tumors like paragangliomas, clear cell sarcomas, alveolar soft part sarcomas (ASPS), and malignant melanomas [19]. The consideration of next generation sequencing may aid in distinguishing between malignant PEComa and metastatic melanoma [20]. While there is currently no established optimal management strategy for PEComa, surgery remains the primary treatment approach [21]. In this study, all 11 patients underwent surgical intervention involving local excision of the lesion. However, for lesions with high-risk characteristics, there is a paucity of clinical evidence supporting the selection and efficacy of postoperative complementary treatment modalities. It is noteworthy that all three patients diagnosed with malignant PEComa in this study received adjuvant therapy following surgery, and none experienced recurrence or metastasis during the final follow-up period. Targeted therapy, particularly mTOR inhibitors, may be considered a viable treatment option for patients with malignant recurrence or distant metastases [22]. A multi-center, open-label, prospective study has provided evidence of the effectiveness of the mTOR inhibitor nab-sirolimus in terms of its rapid therapeutic effects, sustained response, favorable disease control rates, and notable safety profile. Therefore, nab-sirolimus represents a significant novel therapeutic option for the management of malignant PEComa [23].

## Conclusions

The clinical presentation, tumor biomarkers, and imaging features of PEComa exhibit a lack of specificity, requiring dependence on pathological examination and immunohistochemical analysis for precise diagnosis. Surgical resection is the mainstay of treatment, with patients typically demonstrating a favorable prognosis. Due to the rarity of this disease, we have undertaken a thorough review of 11 cases across various anatomical locations to provide a valuable resource for clinical management.

## Data Availability

The datasets underpinning the conclusions of this study can be obtained from the corresponding author upon a reasonable request.
